# Microbiome yarns: microbiomology of winter rosy face^1^^,^ 2 ^,^ 3 ^,^ 4

**DOI:** 10.1111/1751-7915.12711

**Published:** 2017-04-12

**Authors:** Kenneth Timmis, Franziska Jebok

**Affiliations:** ^1^ Institute of MicrobiologyTechnical University BraunschweigBraunschweigGermany; ^2^ Institute for Educational ScienceUniversity of FreiburgFreiburgGermany

*The Microbiome Channel, Studio 7A, BBZ Plaza, Burbank, 7.30 pm: Abigail Repor‐Tastory, Discovery Presenter, turns to face the camera*: Good evening and welcome to a new episode of ‘*Microbiome Discoveries that Change our Lives*’. Our guest this evening is once again Dr. Anastasia Noitall‐Most^5^ from the Streber Elite University of Los Angeles. Good evening Dr. Noital‐Most *(shaking hands)* and thank you for appearing on the programme.

*Dr. Noitall‐Most*: Good evening Abi; it is always a pleasure to be here.

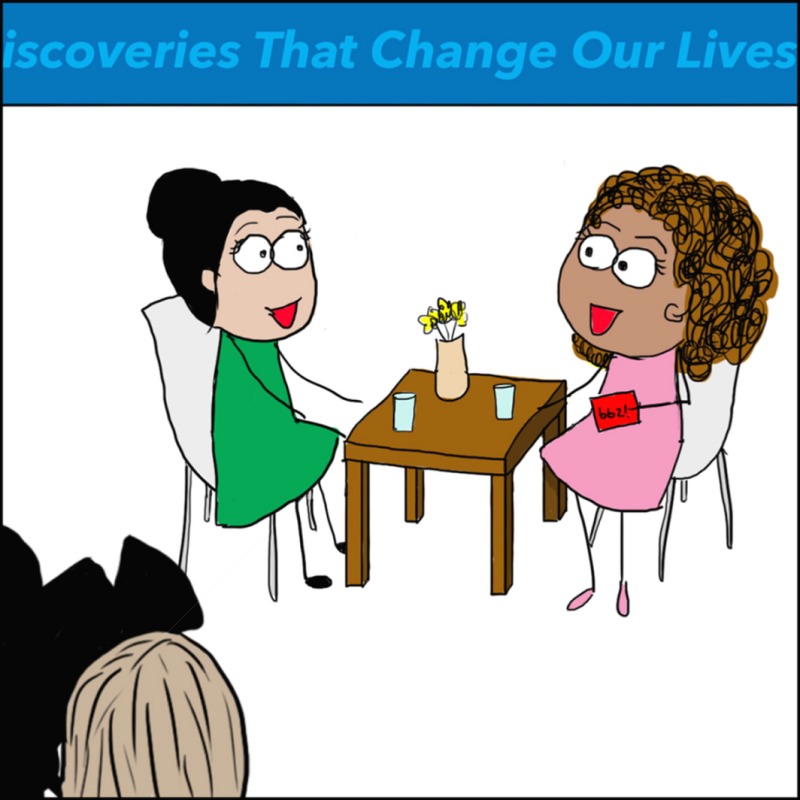

*Ms. Repor‐Tastory*: Ani: this evening we want to discuss what seems to be a very curious phenomenon that was reported recently, namely the hibernation of facial microbes in winter.

*Dr. Noitall‐Most*: Yes, Abi: this is a very curious and unexpected finding by the groups of Professor Scarlett Havabotoxy and Dr. Gotthilf von Pickelface at the European Centre for Clinical Dermatology in Slovenia that was published in Natural Science.

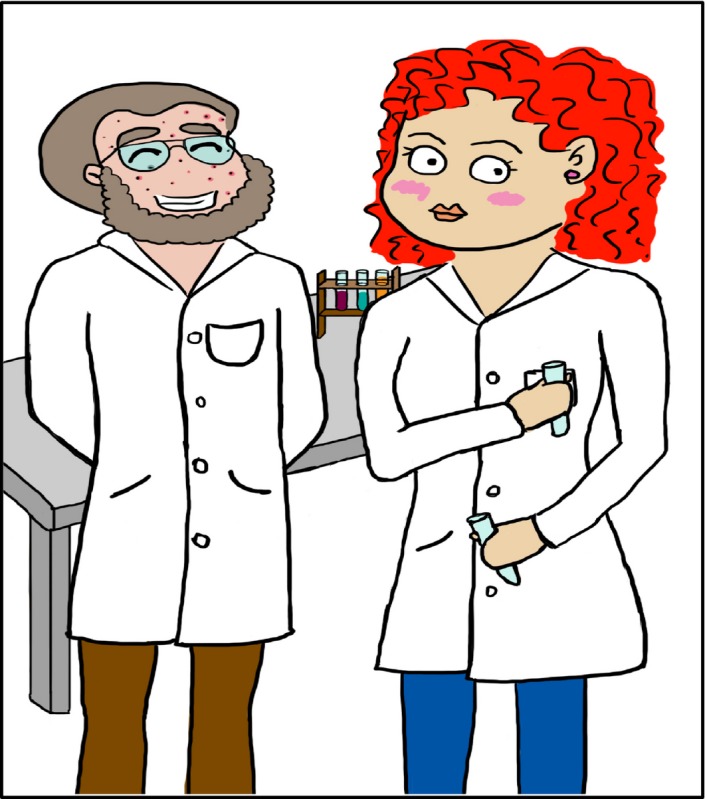

The background to the story is, in essence, when outside temperatures plummet and we turn up the central heating of our houses and workplaces, the exposed parts of our body – primarily our faces – are subjected to two environmental insults, namely cold, often exacerbated by a chill factor and low humidity. The result is the well‐known drying of the facial skin which flakes and becomes red.

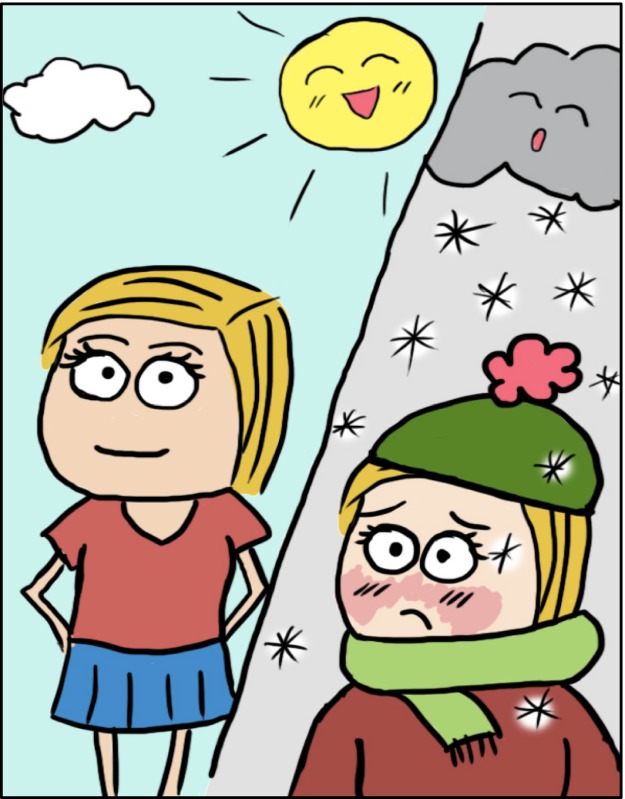

*Ms. Repor‐Tastory:* So, Ani: what is new and how does it relate to our microbiome?

*Dr. Noitall‐Most:* Well, previously, it was assumed that the so‐called winter rosy face, or WRF, which ranges from a bit of flakiness to the *uncooked burger patty* appearance and Rosacea, was due to dryness/cold‐induced damage of the skin and a resulting inflammatory reaction that causes the typical symptoms.

*Ms. Repor‐Tastory, running her fingers absent‐mindedly across her cheeks to assess skin texture under the heavy stage make‐up*: Yes I thought so too, and deal with it by using tons of moisturizers.

*Dr. Noitall‐Most:* Yes, Abi, but these treat the symptoms, not the cause, so only give temporary relief. And anyway, the new skin microbiome results obtained by the ECCD have consigned the old theory to the bin. The experiments carried out were rather interesting. The researchers did a double‐blinded, randomized trial in which groups stratified according to age, gender and skin type were subjected to either a drying atmosphere, a cold, windy atmosphere, a dry, cold windy atmosphere, or a warm moist atmosphere which served as the control. Over a period of two weeks, facial skin surface microbiomes were sampled and their compositions determined. In a nutshell, as expected, the ECCD researchers found that both drying and coldness reproducibly changed global microbiome compositions of the face, qualitatively and quantitatively, and as expected changed the facial microbiome gene expression. However, while there was a broad correlation between average WRF symptoms and microbiota changes, puzzlingly, there was little correspondence at the individual level. So the researchers did so‐called deep sequencing to identify and investigate minor members of the microbiome and came up with something that was unexpected.

*Ms. Repor‐Tastory:* One moment, Ani: we have now had several episodes on the topic of the human microbiome, so our viewers are familiar with it in general terms. But deep sequencing is new: what does it mean?

*Dr. Noitall‐Most*: Oh sorry! A problem with microbiome sequencing is the variation in population sizes of different organisms. So, for example, 1000 cells of microbe A may be present in a sample, whereas only a single cell of microbe B may be present, which may nevertheless play an important role. Limited sequencing of the sample DNA will only reveal the presence of microbe A. To detect microbe B, it is necessary to sequence the material so extensively that microbe A will be detected more than 1,000 times. This is deep sequencing: sequencing to reveal the presence of microbiome members present in low numbers. If such bugs seem to be globally rare, they are called members of the *rare biosphere* 6 and not much is known about them or what they do.

Anyway, to continue the story, when this was done, a new bacterium was discovered in the microbiomes of control faces that had not been seen before, and belongs to the *rare biosphere*. Interestingly, this bug was completely absent from the microbiomes of cold and/or dry skins. However, in a serendipitous discovery following a nurses’ weekend party, and a hung‐over nurse unintentionally sampling the skin microbiome too enthusiastically,

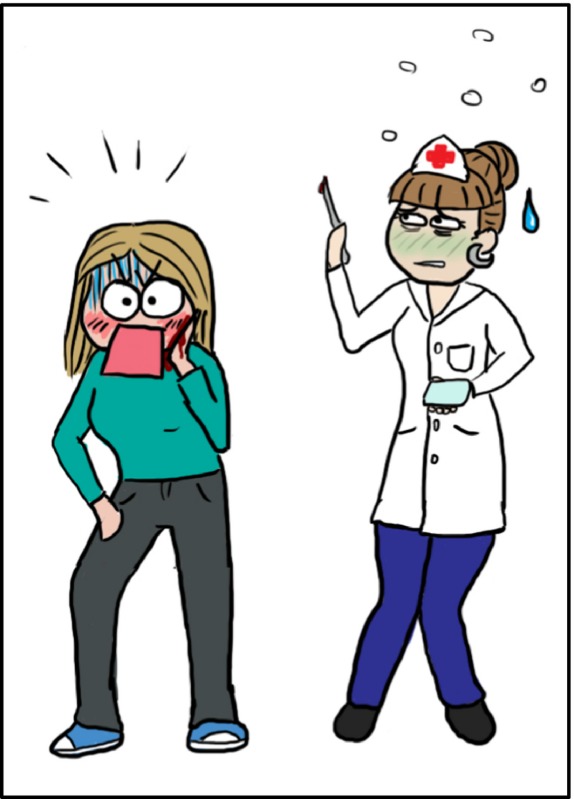

It was discovered that the new bug had not actually disappeared but had instead burrowed into the skin and then essentially gone to sleep – it was not gene expression active! BUT: apparently, its invasion of the skin was sufficiently irritating to trigger an inflammatory response which – hey presto – resulted in WRF!

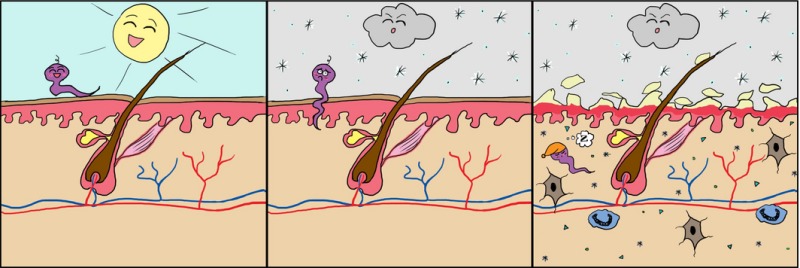

The business of going offline and shutting down metabolism to a minimal level is well known in microbiology as the transition to a dormant or persister state^7^, becoming viable but not culturable^8^, and mutating to a small‐colony variant^9^, and is important for species survival during periods of stress and for counteracting extinctions^10^. In the case of infecting pathogens, the reduced metabolism and growth rate diminish vulnerability to antimicrobial drugs and host defences. This reduces therapy efficacy and may allow survival of small numbers of the infecting pathogen during therapy, such that the disease flares up again once therapy is (apparently successfully) terminated. Such forms are often responsible for chronic infections since their eradication is clinically challenging.

Anyway: back to the story. Since the burrowing bug is related to *Yersinia*, previously called *Pasteurella*, some members of which are pathogens, the ECCD folks called this new bacterium *Burrowsia cosi*, in recognition of the pioneering work of T. W. Burrows^11^ on *Pasteurella pestis*, the causative agent of plague.

*Ms. Repor‐Tastory, delicately fingering her cheeks*: Wow, Ani, this is mind blowing! So we normally have a close relative of the plague bug sitting comfortably sunning itself on our cheeks, calmly watching the world go by, while waiting for winter, when it can give us a blotchy face?

*Dr. Noitall‐Most*: No: that is an exaggeration! Keep in mind that when it is active, it does no harm and it's only when it seeks to get out of the cold, more or less like we do when winter arrives, does it cause a problem and then only passively, since it is effectively in hibernation.

*Ms. Repor‐Tastory:* Ok, Ani: so what does all this mean in terms of dealing with the problem? Is there any hope of a cure for WRF?

*Dr. Noitall‐Most*: Ali: it is a bit too early to say, but we can be optimistic. What we know is that Genghis Khan who, as you are certainly aware, sired hundreds of sons and created a line whose genetic footprint can be found in a good proportion of present‐day men^12^, and the Golden Horde, did not suffer from WRF, despite the fact that winter in the Gobi can be pretty raw.

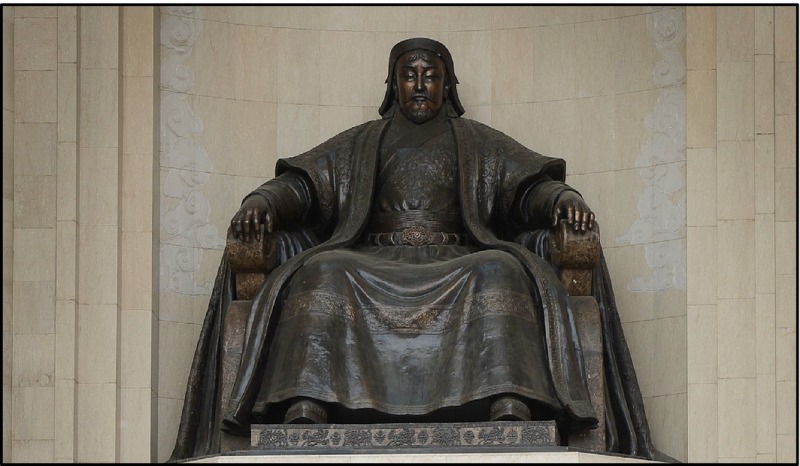

And what we also know about the Golden Horde is that they smeared their faces with sheep fat to minimize cold and wind damage – they did not, of course, have centrally heated yurts at the time. Now that bit of intelligence does not help us directly, because the smell of this particular personal care product was, when it became rancid after a few days, by all accounts bad enough to disable all non‐Mongol enemies within a bow‐shot radius, depending of course on wind direction.

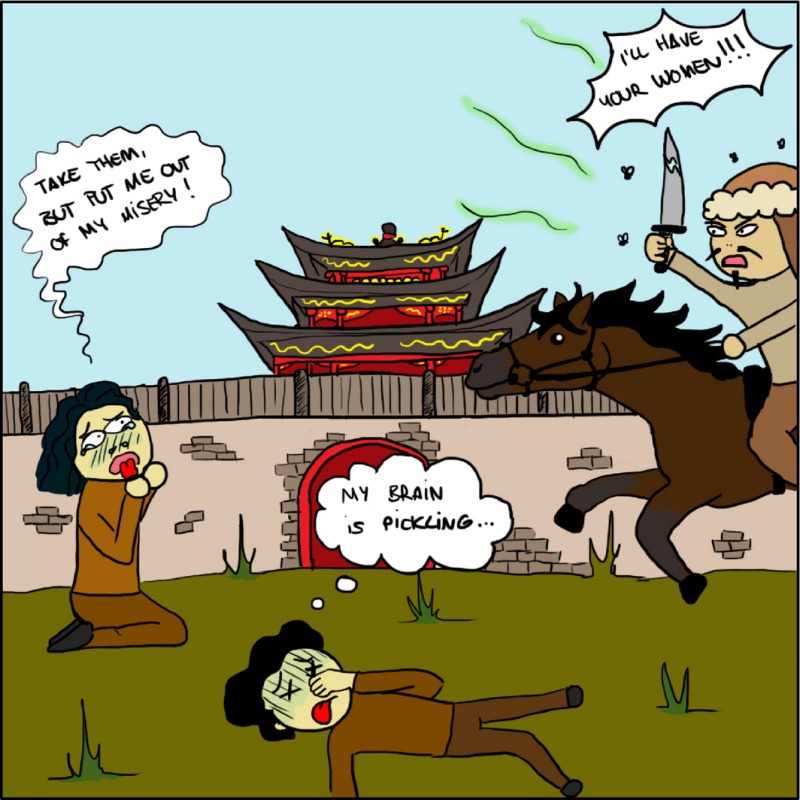

*Ms. Repor‐Tastory, wrinkling her nose and expressing heartfelt disgust*: Uuhhh – how dreadful! The poor women!

*Dr. Noitall‐Most*: Yes, I perfectly agree. However, on the positive side, the sheep fat constitutes a lead, and the ECCD researchers have already found that fat from sheep from the Terelj National Park in Mongolia discourages *Burrowsia cosi* from burrowing.

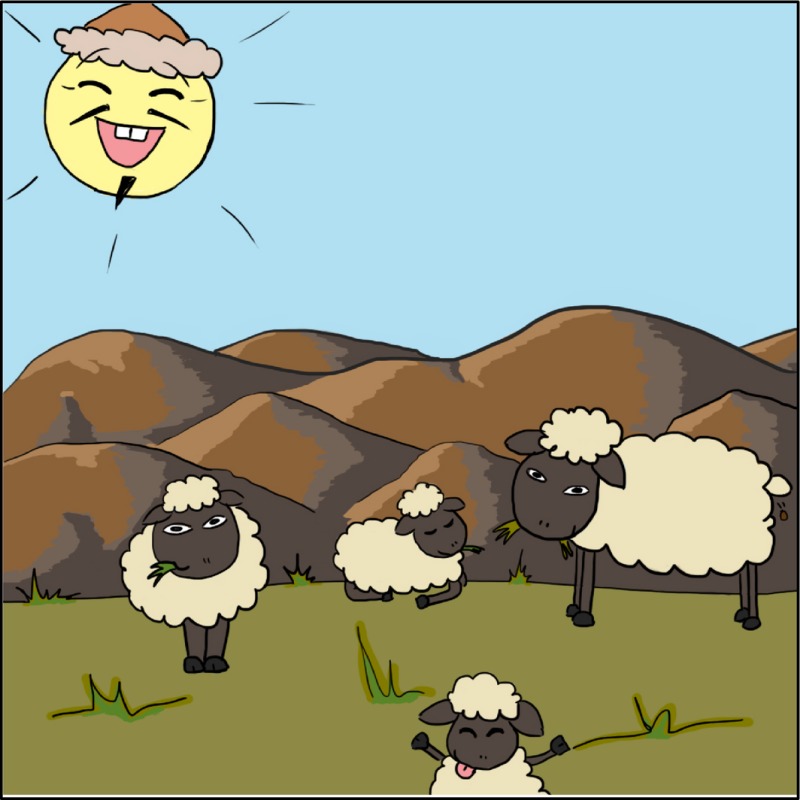

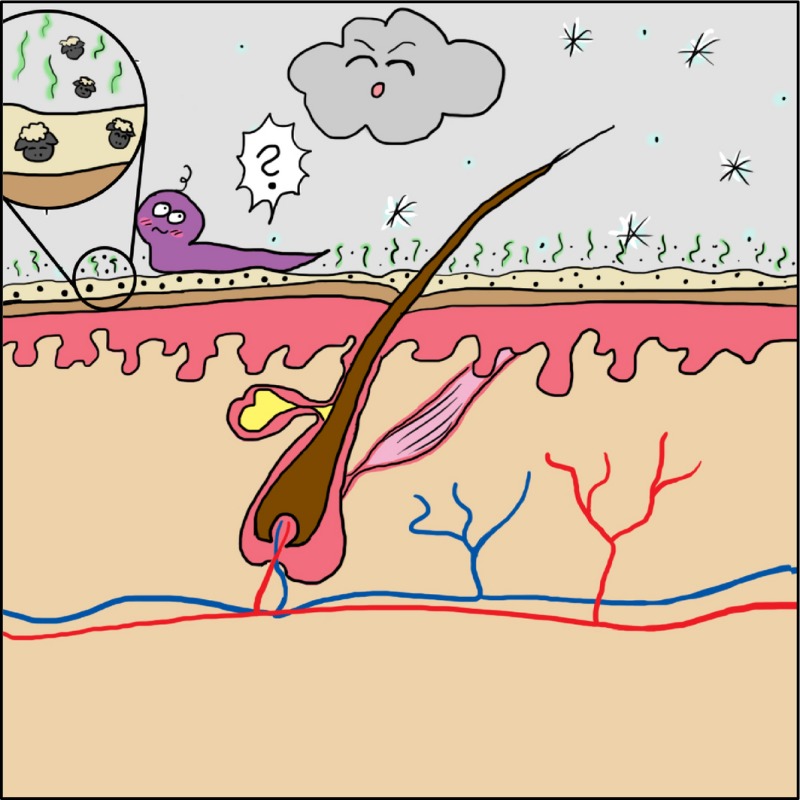

So the race is now on to discover what and how! I have heard that at least one global skincare company is collaborating with experts to analyse the lipids by lipidomics, purify individual lipids and their breakdown products, and test them for their ability to persuade *Burrowsia cosi* not to burrow and go into hibernation. And: from the website of Ovinelipid Skincare (oh, sorry, I did not mean to mention it by name, to avoid seeming to endorse it!), it would appear that at least one compound is effective and is being incorporated into a moisturizing product to be released next winter.

*Ms. Repor‐Tastory, relaxing and looking relieved*: Well: that is really good news! I can see we will need an update on this topic next year. In the meantime, thank you, Ani, for this highly interesting discussion and thank you viewers for joining this episode of ‘*Microbiome Discoveries that Change our Lives*’.

++++++++++++++++++++++++

## Conflict of interest

None declared.

++++++++++++++++++++++++

## Explanatory Notes for Online Version at www.theabsurdmicrobe.com

5 youngest daughter of the renowned Dr. Noitall, see e.g. Koshland, D. E., Jr. 1994. Editorial: A simple proposal for health care rationing. Science **264**: 1515

6 Sogin, M. L., et al. 2006. Microbial diversity in the deep sea and the underexplored ‘rare biosphere’. Proc. Natl. Acad, Sci. USA. 103: 12115–12120

7 Hobby, G. L., et al. 1942. Observations on the mechanism of action of penicillin. Proc. Soc. Exp. Biol. Med. 50: 281–285; for recent reviews, see e.g. Lewis, K. 2010. Persister cells. Annu. Rev. Microbiol. 64: 357–372; Wood, T. K. et al. 2013. Bacterial persister cell formation and dormancy. Appl. Environ. Microbiol. 79: 7166–7121

8 Colwell, R. R. 2000. Viable but noncultural bacteria: a survival strategy. J. Infect. Chemother. **6**: 121–125

9 Proctor, R. A., et al. 1995. Persistent and relapsing infections associated with small‐colony variants of *Staphylococcus aureus*. Clin. Infect. Dis. 20: 95–102; Häussler, S. et al. 1999. Small‐colony variants of *Pseudomonas aeruginosa* in cystic fibrosis. Clin. Infect. Dis. 29: 621–625

10 e.g. Aanderud, et al. 2015. Resuscitation of the rare biosphere contributes to pulses of ecosystem activity. Front. Microbiol. **6**:24. doi: 10.3389/fmicb.2015.00024

11 e.g. Burrows, T. W. 1957. Virulence of *Pasteurella pestis*. Nature **179**: 1246–1247

12 Zerjal, T., et al. 2003. The genetic legacy of the Mongols. Am. J. Human Genet. 72: 717–721

